# Occult Iliac Deep Vein Thrombosis in Second Trimester Pregnancy: Clues on Bedside Ultrasound

**DOI:** 10.5811/cpcem.2017.1.33536

**Published:** 2017-05-09

**Authors:** Roopa Avula, Michael Niemann, Nicole Dorinzi, Kristine Robinson, Melinda Sharon, Joseph Minardi

**Affiliations:** * West Virginia University School of Medicine, Morgantown, West Virginia; † West Virginia University, Department of Emergency Medicine, Morgantown, West Virginia

## Abstract

Isolated pelvic deep vein thromboses (DVT) are rare and difficult to diagnose, but they are more common in pregnant women and carry an increased risk of embolization. Pulmonary embolism is the most common non-obstetric cause of death in pregnancy. Compression ultrasound is the first-line imaging test for suspected lower extremity DVT, but it cannot usually aid in directly visualizing or easily diagnosing isolated pelvic DVT. Nonetheless, point-of-care ultrasound (POCUS) may provide valuable clues to help rule in pelvic DVT and expedite initiation of anticoagulant therapy. Such findings include increased venous diameter, increased resistance to compression, visible venous reflux, and blunted phasicity. This case presents an example of how these findings on POCUS led the emergency physician to make the difficult diagnosis of pelvic DVT at the bedside within seconds.

## INTRODUCTION

Leg pain and swelling are common in pregnancy, which is a risk factor for deep vein thrombosis (DVT).^1^ Isolated pelvic DVT, though uncommon, occurs more often during pregnancy.^2^,^3^ Typically, compression ultrasound is first-line imaging for suspected lower extremity DVT, but it may not as easily identify isolated pelvic DVT unless a more comprehensive sonographic approach and technique is used.^4^

Pulmonary embolism (PE) is the most common non-obstetric cause of death in pregnant women.^3^,^5^ Proximal DVT has a higher likelihood of embolization and mortality than calf DVT; thus, early detection is essential.^6^,^7^

Point-of-care ultrasound (POCUS) for DVT is accurate and widely employed by emergency physicians.^8^,^9^ Typical findings include visualization of thrombus and lack of venous compressibility. For pelvic DVT, findings may not be as clear, but subtle clues may suggest the diagnosis.

We present a case of occult iliac DVT in which lower extremity veins were compressible, but other sonographic clues led to the rapid, accurate diagnosis immediately at the bedside. To our knowledge, this is the first report to describe the detailed POCUS findings that led to this uncommon diagnosis.

## CASE REPORT

A 26-year-old pregnant woman at 23 weeks gestation presented with three days of atraumatic left calf pain. Physical examination revealed circumferential swelling, tenderness, and mild redness to the left calf. Clinical likelihood of DVT was felt to be high and DVT POCUS was performed immediately.

**Image 1A, B:**
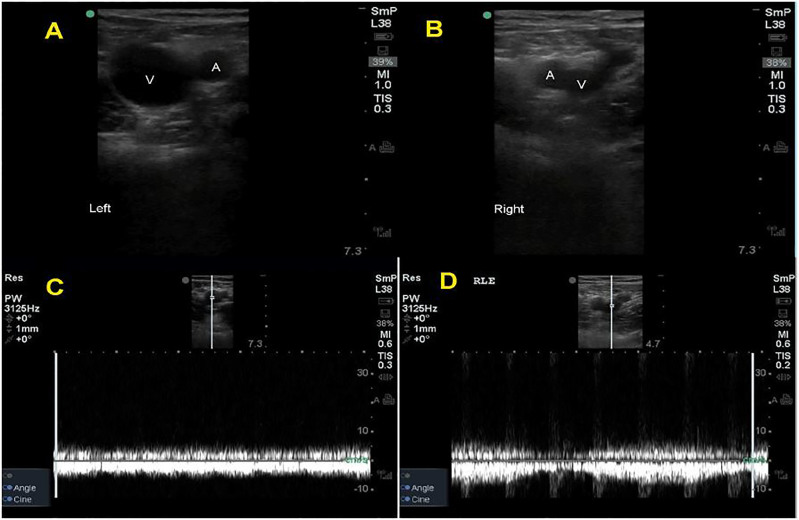
Distended femoral vein. Note the increased diameter of the distended femoral vein (V) in panel A. Compare this to the image from the unaffected side in panel B, where the femoral vein (V) has a much smaller (normal) diameter. (A - artery; V – vein) **Image 1C, D.** Loss of phasicity. Note the flattened waveform in the spectral Doppler tracing in panel C, which represents a loss of normal variation during respiration known as phasicity. Compare this with panel D, which shows gradual changes in velocity of the spectral Doppler waveform corresponding to passive respiration, suggesting patency of the proximal vein. Panel D shows normal phasicity.

**Image 2A, B:**
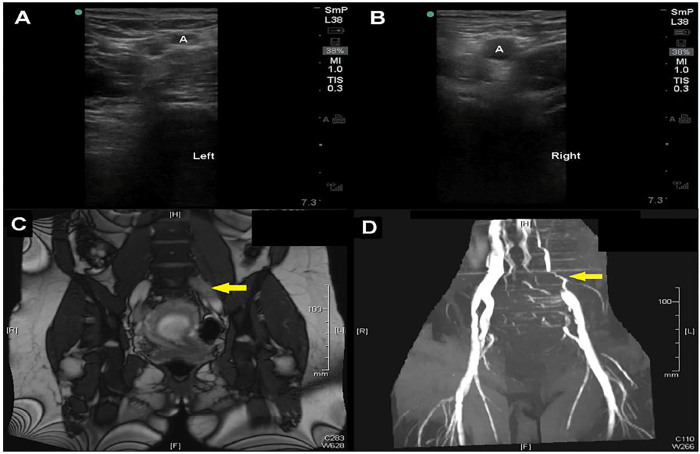
Difficulty in compression. In panel A, venous compression is only achieved with high pressure, as noted by the compressed artery (A). Compare this to panel B, where the vein is completely compressed while the artery (A) is able to maintain a normal diameter. **Image 2C, D.** Common Iliac thrombosis on magnetic resonance: venogram. In panel C, a thrombus is seen as a hypointense signal in the left common iliac vein (arrow). This finding, along with evidence of occlusion, is also evident in panel D (arrow).

Venous compression was performed including views of the proximal zone from the saphenofemoral junction to the common and deep femoral junctions, as well as the popliteal zone to the trifurcation. The deep veins were compressible in all zones but appeared distended and required higher than expected pressure to compress (Images 1A, 1B, 2A, and 2B; Supplemental video). These findings prompted comparison views and additional investigation with pulsed-wave spectral Doppler, revealing normal augmentation but blunted phasicity (Images 1C and 1D; Supplemental video). Deep veins of the unaffected leg were smaller in diameter and compressed more easily (Images 1A, 1B, 2A, and 2B; Supplemental video). These findings suggested a proximal venous thrombus on the left, and anticoagulation was initiated.

A magnetic resonance venogram (MRV) of the pelvis was performed, confirming a near-occlusive thrombus in the left common iliac vein with extension into the external iliac vein, suggestive of May-Thurner syndrome, an anatomical variant where the left iliac vein is compressed by the right iliac artery (Images 2C and 2D).^10^ POCUS findings were referenced by radiology.

The patient was admitted, continued therapy and her course was otherwise uneventful.

## DISCUSSION

DVT is a common consideration for emergency physicians treating pregnant patients with lower extremity symptoms. In pregnant women, proximal DVT is more likely (62%) than calf DVT (6%) compared to the non-pregnant population where 80% of DVTs occur in the calf.^11^ The likelihood of fatal PE is considerably higher when proximal DVT is the source.^6^

Ultrasound for lower extremity DVT is generally accurate; however, its accuracy in cases of isolated pelvic DVT is not well established. In this case, although a clot was not directly visualized, there were clues on POCUS that suggested the diagnosis. These findings included the following:

1. Venous distention (increased diameter and difficulty in compression, specifically compared to the unaffected side)
2. Blunted phasicity (again, specifically compared to the unaffected side)
3. Visible venous reflux.

There is variability in the emergency medicine literature regarding the exact technique of DVT POCUS. We endorse a more comprehensive exam such as that described in this patient. Lack of compressibility suggests DVT, even if a clot is not directly visualized. The Doppler techniques commonly employed include these two:

1. Augmentation – the Doppler gate is placed within the vein of interest; the vein is squeezed distally to assess for a rapid increase in venous velocity, which suggests distal patency.
2. Phasicity – the Doppler gate is placed within the vein of interest and variation during respiration is observed. Blunted phasicity suggests proximal occlusion.

It should be noted that any patient with a high clinical likelihood of DVT and a negative ultrasound should undergo further diagnostic testing, such as computed tomography or MRV. As seen in this case, suggestive findings on POCUS may be adequate to initiate therapy until definitive imaging is obtained.

CPC-EM Capsule

What do we already know about this clinical entity?

POCUS is commonly used by EPs to evaluate for lower extremity DVT. Isolated pelvic DVT is more difficult to identify, but carries a higher risk of embolization.

What makes this presentation of disease reportable?

This report details POCUS findings that may suggest the difficult diagnosis of isolated pelvic DVT.

What is the major learning point?

Increased venous diameter, difficulty in compression, and blunted phasicity may suggest pelvic DVT. Comparison to the unaffected extremity is useful.

How might this improve emergency medicine practice?

Recognition of these findings may suggest an otherwise difficult diagnosis and expedite treatment decisions.

## CONCLUSION

Current literature endorses a two-point focused lower extremity compression examination for DVT, but we endorse a more comprehensive exam.^8^ Pelvic DVTs are rarely identified in the emergency department (ED), and knowledge and attention to these techniques can help expedite diagnosis and management of this dangerous condition. A search of the literature identified only one other case of this diagnosis being made with POCUS in the ED, and the detailed findings were not as thoroughly described.^12^ Our case illustrates use of a more comprehensive approach and suggestions for technique in using POCUS to diagnose pelvic DVT in the emergency setting. We present a case of a young woman in her second trimester of pregnancy with unilateral leg pain and swelling. Though lower extremity veins were compressible, careful attention to subtle clues on DVT POCUS led to the rare and difficult diagnosis of iliac DVT.

Our case demonstrates important teaching points that the use of POCUS in the ED to diagnose isolated pelvic DVT requires a more comprehensive approach and attention to technique that are not widely described in the current literature. The primary teaching points for any questionable cases are as follows:

- Perform comparison views to the unaffected extremity
- Assess for venous distention and compressibility
- Assess for reflux and phasicity

The findings that may suggest pelvic DVT include these:

- Increased venous diameter
- Resistance to compression
- Venous reflux
- Blunted phasicity.

Further, we recommend routinely performing a more detailed DVT POCUS exam as described above in all patients suspected to have a lower extremity DVT.

This case demonstrates the utility of a comprehensive approach and specific details on technique for POCUS in making rapid, accurate diagnoses. Recognition of subtle findings as described here can lead to the uncommon but potentially life-saving diagnosis of isolated pelvic DVT in the ED setting.

## Supplementary Information

Supplemental Video Iliac DVT Findings. In this narrated video, the findings that led to the diagnosis of iliac DVT are demonstrated. Findings include increased venous diameter, difficulty in compression, and loss of phasicity.This ultrasound video clip, performed by the emergency physician, demonstrates a sub-pleural consolidation with surrounding asymmetric B-lines, consistent with pneumonia.
